# A Generalized Bayesian Stochastic Block Model for Microbiome Community Detection

**DOI:** 10.1002/sim.10291

**Published:** 2025-01-24

**Authors:** Kevin C. Lutz, Michael L. Neugent, Tejasv Bedi, Nicole J. De Nisco, Qiwei Li

**Affiliations:** ^1^ Peter O'Donnell Jr. School of Public Health The University of Texas Southwestern Medical Center Dallas Texas; ^2^ Department of Biological Sciences The University of Texas at Dallas Richardson Texas; ^3^ Department of Mathematical Sciences The University of Texas at Dallas Richardson Texas; ^4^ Department of Urology The University of Texas Southwestern Medical Center Dallas Texas

**Keywords:** Bayesian stochastic block model, community detection, Markov random field, microbiome co‐occurrence network, taxonomic tree

## Abstract

Advances in next‐generation sequencing technology have enabled the high‐throughput profiling of metagenomes and accelerated microbiome studies. Recently, there has been a rise in quantitative studies that aim to decipher the microbiome co‐occurrence network and its underlying community structure based on metagenomic sequence data. Uncovering the complex microbiome community structure is essential to understanding the role of the microbiome in disease progression and susceptibility. Taxonomic abundance data generated from metagenomic sequencing technologies are high‐dimensional and compositional, suffering from uneven sampling depth, over‐dispersion, and zero‐inflation. These characteristics often challenge the reliability of the current methods for microbiome community detection. To study the microbiome co‐occurrence network and perform community detection, we propose a generalized Bayesian stochastic block model that is tailored for microbiome data analysis where the data are transformed using the recently developed modified centered‐log ratio transformation. Our model also allows us to leverage taxonomic tree information using a Markov random field prior. The model parameters are jointly inferred by using Markov chain Monte Carlo sampling techniques. Our simulation study showed that the proposed approach performs better than competing methods even when taxonomic tree information is non‐informative. We applied our approach to a real urinary microbiome dataset from postmenopausal women. To the best of our knowledge, this is the first time the urinary microbiome co‐occurrence network structure in postmenopausal women has been studied. In summary, this statistical methodology provides a new tool for facilitating advanced microbiome studies.

**Abbreviations**
ARI: adjusted Rand index
BIC: Bayesian information criterion
MAP: maximum a posteriori
MCLR: modified centered‐log ratio
MCMC: Markov chain Monte Carlo
MRF: Markov random field
rUTI: recurrent urinary tract infection
SBM: stochastic block model

## 1 Introduction

The term *microbiome* was first introduced by Nobel laureate Joshua Lederberg and refers to the collective genomes of microorganisms or the microorganisms themselves [1, 2, 3]. Ecological interactions of these microorganisms are important because they affect microbiome function and host health through the formation of complex microbiome communities [4]. Uncovering these relationships is essential to understanding the role of the microbiome in disease progression and susceptibility [5, 6]. For example, microbial interactions in the human gut microbiome have been associated with the progression of several diseases such as colorectal cancer [7], diabetes [8], and inflammatory bowel disease [9]. Hall et al. [5] found evidence that the network of microbes in the human gut microbiome is composed of distinct communities that co‐occur and interact with one another. Further, it was found that each community tends to have similar metagenomic functional properties. Thus, uncovering the underlying community structure of a microbiome network is the key to understanding its impact on human health [10]. From this point forward, a network of microbes will be referred to as a *microbiome co‐occurrence network* and is characterized by whether or not the microbes associate with one another.

*Network analysis* is a field where a variety of methods are used to infer the complex structure and associations among network entities such as persons or microbes [11]. A network is modeled as a graph, which is a set of nodes and edges. In microbiome research, each node is a taxon and an existing edge represents a significant association between two taxa. An inferred microbiome co‐occurrence network can help characterize taxon‐taxon associations and reveal their latent properties, mechanisms, and structures [12]. Co‐occurrence research within microbiomes usually considers taxon‐taxon associations, which means all microbes are from the same level of the taxonomic tree hierarchy [13]. Network analysis methods use either a similarity metric or a model‐based approach to determine these associations. For further information, Lutz et al. [13] provide a survey of available methods for microbiome network analysis.

*Community detection* is one of the fundamental problems in network analysis. A community is characterized by nodes that are densely connected to one another within that community but are sparsely connected to nodes in other communities [14]. The stochastic block model (SBM) is one of several methodological approaches to community detection. Holland et al. [15] were the first to introduce the mathematical foundations of SBM. However, Guimerà et al. [16] were, to our knowledge, the first to use the SBM to perform model selection to estimate network structure. In general, SBMs are probabilistic models that aim to cluster the nodes (e.g., taxa) of a network with heterogeneous connectivity patterns into mutually exclusive blocks with homogeneous connectivity patterns [17, 18, 19]. This clustering property presents a natural way to detect communities in a network [20]. Furthermore, SBMs can infer the latent underlying structural patterns of a network and estimate the edge probabilities within each block (intra‐block) and between blocks (inter‐block) [16]. SBMs have been employed in a broad range of applications including medicine [21], social media [22], sociology [17], political science [23], military strategy [24], infrastructure [25], and many more. Applications in microbiome co‐occurrence networks of bacteria, genes, or proteins include microbial communities associated with pH in arctic soil [26], taxon‐taxon communities of the human gut microbiome associated with disease development [5, 27], protein‐protein interactions associated with pancreatic cancer [28], optimizing treatment plant operations by understanding microbial communities in wastewater [29], controlling tick‐related diseases by understanding the interactions of tick‐borne microbial communities over time [30], and the use of protein‐protein communities related to SARS‐CoV‐2 to better understand COVID‐19 [31]. Recently, SBMs have been used to uncover the underlying microbiome community structure by clustering all taxa in the microbiome co‐occurrence network based on their connectivity patterns [5, 32, 33]. Applications using frequentist [22, 23, 28, 34, 35], Bayesian [5, 17, 36, 37, 38, 39], and algorithmic [40, 41, 42, 43] approaches to clustering are available in the literature. Some approaches can leverage nodal covariates in addition to the network information [35, 38, 42, 43], which may improve community detection. For example, a class of extended stochastic block models (ESBM) [38] offers a nonparametric Bayesian mixture model using generic Gibbs‐type priors (e.g., Dirichlet‐multinomial, Dirichlet process, Gnedin process, and Pitman‐Yor process) for clustering an undirected binary network while leveraging multiple categorical nodal covariates. Also, sbm‐cov [35] offers a model‐based spectral algorithm for clustering an undirected binary network while leveraging one categorical nodal covariate. These methods encourage two nodes to be in the same community if their respective covariates are equal. Table S1 in the Supporting Information provides a non‐exhaustive catalog of available SBM software package information for users in R, Python, and C++, while Table S2 lists the websites for software access and documentation.

We searched the literature for specific applications of microbiome network analysis [5, 28, 33, 44, 45, 46, 47, 48, 49, 50], and found that none of them account for taxonomic tree structure. Integrating the available taxonomic or phylogenetic tree structure could provide additional information about microbiome communities because taxa (e.g., species) having the same parent (e.g., genus) or with similar functional properties tend to cluster together [5, 51, 52, 53]. In addition, most of these applications ignore multiple characteristics of taxonomic abundance data generated from metagenomic sequencing technology [54] such as high‐dimensionality, zero‐inflation, over‐dispersion, and compositionality [55, 56], resulting in information loss and inference bias [57]. Thus, tailored statistical methods accounting for those challenging characteristics are required to perform community detection on a microbiome co‐occurrence network.

This article proposes a generalized Bayesian SBM with a Markov random field (MRF) prior, which we refer to as Bayesian‐SBM‐MRF. The MRF prior accounts for taxonomic tree structure by incorporating more than one level of the taxonomic tree, which is an attractive feature of our proposed model as well as a novel use of the prior. The choice of prior is the main difference between our model and ESBM. Further, we employ a parametric Bayesian model while ESBM employs a nonparametric Bayesian model. Our approach also accounts for zero‐inflation and compositionality, unlike sbm‐cov and ESBM, by normalizing the taxonomic abundance data using the recently developed modified centered‐log ratio (MCLR) transformation [58] as a data preparation step. Next, our proposed model considers two sources of binary information to perform microbiome community detection: (i) the undirected taxon‐taxon microbiome co‐occurrence network and (ii) taxonomic tree information (i.e., nodal covariates). Our SBM is a parametric Bayesian finite mixture model that depends on two binary matrices to incorporate (i) and (ii). We use the Spearman correlation coefficient of the MCLR‐transformed abundances to identify significant non‐linear pairwise taxon‐taxon associations as a way to estimate (i). Additionally, the matrix for (ii) indicates whether two nodes have the same parent, which our model leverages *via* the MRF prior. We show in our simulation study that Bayesian‐SBM‐MRF performs at least as good if not better when we incorporate both (i) and (ii) instead of just only (i) in the analysis, and significantly outperforms other existing SBMs and clustering algorithms. We implement our model on a real urinary microbiome dataset taken from a controlled, cross‐sectional study of recurrent urinary tract infections (rUTI) from postmenopausal women. To the best of our knowledge, this is the first time that the urinary microbiome community structure has been investigated with respect to rUTI from postmenopausal women. The results of our analysis provide the foundation for future studies related to the urinary microbiome and human health. Our model provides a new powerful tool for advanced studies of community detection in microbiome co‐occurrence networks.

The remainder of the article is organized as follows: Sections 2 and 3 introduce the data preprocessing, the standard Bayesian SBM, and the proposed generalized SBM with an MRF prior; Section 4 describes the Markov chain Monte Carlo (MCMC) algorithms for model fitting and Bayesian posterior inference; Section 5 provides the results from the analysis on a real urinary microbiome dataset as well as the results of a simulation study to assess and compare the proposed model to competing methods; Section 6 concludes the article with a summary and discussion of the proposed Bayesian‐SBM‐MRF model.

## 2 Data Preparation

**FIGURE 1:**
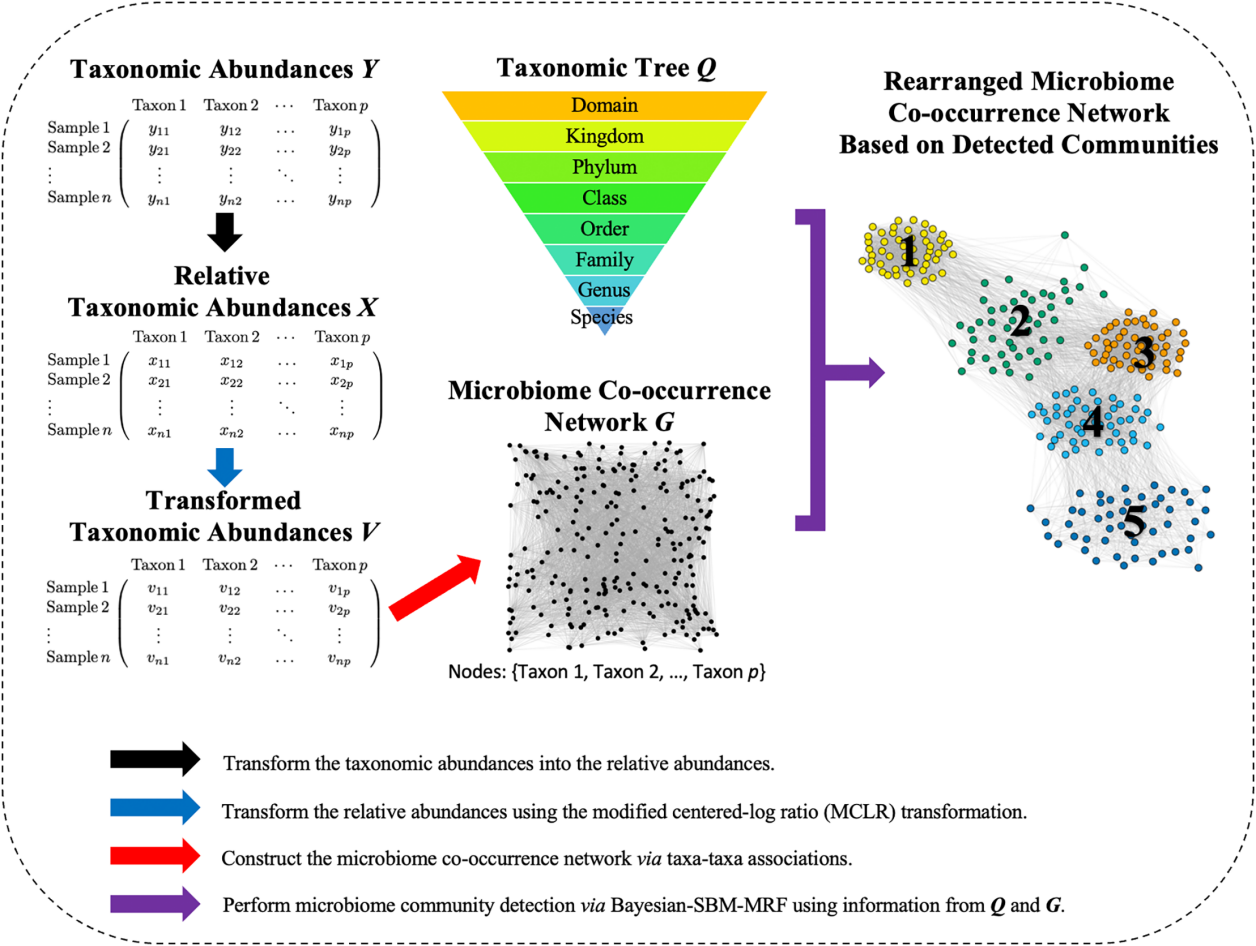
An illustration of the Bayesian‐SBM‐MRF workflow. The taxonomic abundance data Y, which are generated from metagenomic sequencing, contain n samples and p taxa. Data preparation is a three‐step procedure. First, row‐wise normalization is applied to Y to transform the abundances into the relative abundances X (black arrow). Second, the modified centered log‐ratio (MCLR) transformation is applied to X, which results in matrix V (blue arrow). Third, the microbiome co‐occurrence network G with p nodes is estimated from pairwise associations of the transformed taxonomic abundance data V (red arrow). Then, community detection is performed using Bayesian‐SBM‐MRF to infer the underlying microbiome community structure using information from both the microbiome co‐occurrence network G and the taxonomic tree information Q (purple arrow). [Colour figure can be viewed at wileyonlinelibrary.com]

In this section, we describe the two sources of binary taxonomic information to perform microbiome community detection; namely, the microbiome co‐occurrence network and taxonomic tree, as illustrated in Figure 1.

Let $Y=[y_{ij}]\in {\mathbb{N}}^{n\times p}$ denote an n×p taxonomic abundance matrix, with $y_{ij}$ indicating the count of taxon j observed from sample i where i=1,…,n and j=1,…,p. We use $y_{i\cdot }={\left(y_{i1},\ldots ,y_{ip}\right)}^{⊤}$ and $y_{\cdot j}={\left(y_{1j},\ldots ,y_{nj}\right)}^{⊤}$ to denote the vector from the $i^{\text{th}}$ row and $j^{\text{th}}$ column of Y, respectively. We use the same formatting for any matrix throughout this article.

### 2.1 Microbiome Co‐Occurrence Network

Let $X=[x_{ij}]\in {\left[0,1\right]}^{n\times p}$ denote the n×p matrix of the relative abundances (i.e., compositions), where $x_{ij}=y_{ij}/\sum_{j=1}^{p}y_{ij}$. The vector of relative abundances in the $i^{\text{th}}$ sample, $x_{i\cdot }$, is defined on the p‐dimensional simplex (i.e., $x_{ij}\geq 0,\forall j$ and $\sum_{j=1}^{p}x_{ij}=1$). To map a composition to a Euclidean vector space, Aitchison [59] proposed the CLR transformation, which is to scale the relative abundances $x_{i\cdot }$ by their geometric mean and then take the logarithm to remove the unit‐sum constraint. However, taxonomic abundance data contain a large proportion of zero counts attributed to rare or low‐abundance taxa that may be present in only a small percentage of samples; whereas, others are not recorded due to the limitations of the sampling effort. The CLR transformation adds an arbitrary pseudo value to both non‐zero and zero values, which disguises the zeros and may lead to spurious correlations between taxa because zeros and non‐zeros are treated equally. To remedy this issue, Yoon et al. [58] recently proposed MCLR, which transforms only the non‐zero values, and they demonstrated that MCLR reduces bias and variance compared to CLR. Specifically, the MCLR transformation, given by Equation (1), takes the log of the ratio of each $x_{ij}\neq 0$ and the geometric mean of all non‐zeros in $x_{i\cdot }$. Let $V=[v_{ij}]\in {\mathbb{R}}^{n\times p}$ denote the n×p matrix of the MCLR‐transformed relative abundances, each element of which is expressed as

(1) $$v_{ij}=\left\{\begin{array}{l} \begin{array}{ll} 0 & \text{if}\;x_{ij}=0\\ \log(x_{ij}/\tilde{g}(x_{i\cdot }))+\epsilon_{i} & \text{if}\;x_{ij}\neq 0 \end{array} \end{array}\right.$$

where $\tilde{g}(x_{i\cdot })={\left(\prod_{j=1}^{p}x_{ij}^{I(x_{ij}\neq 0)}\right)}^{1/\sum_{j=1}^{p}I(x_{ij}\neq 0)}$ gives the geometric mean of the non‐zero relative abundances in the i‐th sample, and I(·) is the indicator function. Note that MCLR reduces to robust CLR [60] when $\epsilon_{i}=0,\forall i$. Alternatively, we can make all non‐zero entries strictly positive by letting $\epsilon_{i}=1+|\min_{\left\{j:x_{ij}\neq 0\right\}}\log(x_{ij}/\tilde{g}(x_{i\cdot }))|$ as suggested by Yoon et al. [58] who impose a shift above zero on the transformed values.

While the log transformation is a feature of MCLR, it does not guarantee linearization [61], especially since the transformation only applies to each $x_{ij}\neq 0$. The data remain positively skewed after transformation due to the non‐transformed zeros. As a result, to measure the similarity between any pair of taxa j and $j^{\prime }$ (where $j^{\prime }=1,\ldots ,p$) in terms of MCLR‐transformed relative abundances (i.e., between $v_{\cdot j}$ and $v_{\cdot j^{\prime }}$), we use their Spearman correlation coefficient $\rho_{jj^{\prime }}$ to account for non‐linearity [62]. We further test the null hypothesis $\rho_{jj^{\prime }}=0$ versus the alternative $\rho_{jj^{\prime }}\neq 0$ and obtain a p‐value to determine the significance. All p‐values are further adjusted using the Benjamini‐Hochberg procedure to control the false discovery rate [63]. To construct the microbiome co‐occurrence network represented by a p×p binary adjacency matrix $G=[g_{jj^{\prime }}]\in {\left\{0,1\right\}}^{p\times p}$, we assigned an edge as $g_{jj^{\prime }}=1$ if the corresponding adjusted p‐value of $\rho_{jj^{\prime }}$ is less than the significance level of α=0.05 and zero otherwise. The existence of an edge represents a significant association between taxa j and $j^{\prime }$; whereas, the absence of an edge represents no significant association between taxa j and $j^{\prime }$. We also assume no self‐loops in G, so $g_{jj^{\prime }}=0$ when $j=j^{\prime }$.

Even though one may argue that binarizing G results in some information loss, experts agree that very little information is lost when sequencing data are dichotomized [64]. In fact, sequencing data suffer from many biological and technical errors that make these data very noisy [65]. Actually, dichotomization is not uncommon practice and helps to reduce noise in sequencing data, which has produced reasonable results [64, 66, 67, 68, 69]. Further, Steinway et al. [70] found that information loss due to dichotomization of their gut microbiome network data was negligible.

### 2.2 Taxonomic Tree

We make use of taxonomic tree information by indicating if two taxa (e.g., species) have the same parent (e.g., genus). Let $Q=[q_{jj^{\prime }}]\in {\left\{0,1\right\}}^{p\times p}$ denote a p×p binary matrix where $q_{jj^{\prime }}=1$ indicates that taxa j and $j^{\prime }$ have the same parent and zero otherwise for $j\neq j^{\prime }$. Note that Q is not the adjacency matrix of the taxonomic tree, but rather of the graph representing whether two taxa share a parent in the taxonomic tree. We assume that no taxon is its own parent, so we set $q_{jj^{\prime }}=0$ when $j=j^{\prime }$. Matrix Q allows the model to include the taxonomic tree information, which is an attractive feature of our model. As a caution, we recommend incorporating only genus or family level at most as parents. If you incorporate information too far up the taxonomic tree, then all the taxa will naturally collapse into one group, rendering useless results.

## 3 Model

Here, we first review the standard Bayesian SBM model in Section 3.1 and then provide the full details about our proposed Bayesian‐SBM‐MRF in Section 3.2.

### 3.1 A Review of the Bayesian Stochastic Block Model

Our model is a generalized form of the standard Bayesian SBM for an undirected binary network [17, 71], which specifically is a finite Bernoulli mixture model with K components, where K is fixed at a prespecified value. We discuss how we determined the optimal value of K for our model in Section 4. Further, the standard Bayesian SBM for an undirected binary network depends on the community membership vector z and the edge probability matrix Ω. The parameters z and Ω are independent by assumption. Let $z=[z_{j}]\in {\left\{1,\ldots ,K\right\}}^{p\times 1}$ denote the community labels for taxon j=1,…,p, where $z_{j}=k$ indicates that taxon j belongs to community k for k=1,…,K. The total number of taxa belonging to community k is denoted by $n_{k}=\sum_{j=1}^{p}I(z_{j}=k)$. Next, $\Omega =[\omega_{kk^{\prime }}]\in {\left[0,1\right]}^{K\times K}$ contains the edge probabilities, where each diagonal element $\omega_{kk}$ and each off‐diagonal element $\omega_{kk^{\prime }}$ (for $k\neq k^{\prime }$ and $k^{\prime }=1,\ldots ,K$) indicate the probability of observing an edge between any two taxa within community k and an edge between any two taxa between communities k and $k^{\prime }$, respectively.

An edge between taxa j and $j^{\prime }$ is denoted as $g_{jj^{\prime }}$ and is considered to be random. We assume that an edge $g_{jj^{\prime }}$ is a Bernoulli random variable (since our network is binary) and exists with probability $\omega_{kk^{\prime }}$ conditional on taxon j belonging to community k and taxon $j^{\prime }$ belonging to community $k^{\prime }$. This relationship is expressed as

$$g_{jj^{\prime }}|z_{j}=k,z_{j^{\prime }}=k^{\prime },\omega_{kk^{\prime }}∼\text{Bern}(\omega_{kk^{\prime }}),$$

and edges are conditionally independent. Thus, we can express the full data‐likelihood as

$$\begin{array}{ll} f(G|z,\Omega ) & =\underset{k\leq k^{\prime }}{\prod }\;\underset{\left\{j<j^{\prime }:z_{j}=k,z_{j^{\prime }}=k^{\prime }\right\}}{\prod }\text{Bern}(g_{jj^{\prime }}|\omega_{kk^{\prime }})\\ & =\underset{k\leq k^{\prime }}{\prod }\omega_{kk^{\prime }}^{M_{kk\prime }}{\left(1-\omega_{kk^{\prime }}\right)}^{N_{kk\prime }-M_{kk^{\prime }}}, \end{array}$$

where $M_{kk^{\prime }}$ and $N_{kk^{\prime }}$ are the number of observed and total possible edges, respectively, within community k (when $k=k^{\prime }$) or between communities k and $k^{\prime }$ (when $k\neq k^{\prime }$). Also, there are no self‐loops in G as previously mentioned in Section 2.1. Specifically,

Nkk′=nk2ifk=k′nknk′ifk≠k′andMkk′=∑j=1p∑j′=1pI(gjj′=1)I(zj=k)I(zj′=k′).

Latent variables, particularly in Bayesian methodology, are model parameters such as community membership that must be inferred using a statistical model [72]. We model the community membership for each taxon, $z_{j}$, as latent variables from a multinomial distribution, which is expressed as

(2) $$z_{j}|\pi ∼\text{Mult}(1,\pi )$$

where $\pi ={\left(\pi_{1},\ldots ,\pi_{K}\right)}^{⊤}$, $\sum_{i=1}^{K}\pi_{i}=1,$ and $\pi_{k}\in [0,1]$ gives the probability of j belonging to community k *a priori*. Next, π is assumed to be a random variable. So, we place a Dirichlet prior on π, that is, π∼Dirichlet(α), where $\alpha ={\left(\alpha_{1},\ldots ,\alpha_{K}\right)}^{⊤}$ is a positive real‐valued vector. Each $\alpha_{k}$ is usually set to 1 to obtain a uniform hyperprior [73]. Thus, the full conditional posterior density for taxon j belonging to community k (i.e., $z_{j}=k$) given everything else can be expressed as

(3) $$\begin{array}{ll} p(z_{j}=k|z_{-j},\Omega ,G) & \propto f(G|z,\Omega )p(z_{j}=k,z_{-j}|\pi )p(\pi )\\ & =\underset{k\leq k^{\prime }}{\prod }\omega_{kk^{\prime }}^{M_{kk^{\prime }}}{\left(1-\omega_{kk^{\prime }}\right)}^{N_{kk^{\prime }}-M_{kk^{\prime }}}\frac{n!}{n_{1}!\cdots n_{K}!}\pi_{k}^{n_{k}} \end{array}$$

where $z_{-j}$ denotes all the elements in z excluding the $j^{\text{th}}$ element. Let $\eta ={\left(\eta_{1},\ldots ,\eta_{K}\right)}^{⊤}$ denote the normalized posterior probability vector for community membership, where $\eta_{k}=p(z_{j}=k|z_{-j},\Omega ,G)/\sum_{m=1}^{K}p(z_{j}=m|z_{-j},\Omega ,G)$. Specifically, $\eta_{k}$ is the normalized probability that taxon j belongs to community k given the membership of all the other taxa, and the matrices Ω and G. Then, we can sample each community membership label, $z_{j}$, from a multinomial distribution

$$z_{j}|z_{-j},\Omega ,G∼\text{Mult}(1,\eta ).$$

Finally, independent beta priors are imposed on each $\omega_{kk^{\prime }}$, which is expressed as $\omega_{kk^{\prime }}∼\text{Beta}(a_{\omega },b_{\omega })$ where $a_{\omega }\;\text{and}\;b_{\omega }$ are fixed hyperparameters. A common non‐informative setting of this prior is $a_{\omega }=b_{\omega }=1$ [73]. This conjugate prior results in a beta posterior distribution: $\omega_{kk^{\prime }}|z,G∼\text{Beta}(M_{kk^{\prime }}+a_{\omega },N_{kk^{\prime }}-M_{kk^{\prime }}+b_{\omega })$.

### 3.2 Generalized Bayesian Stochastic Block Model With a Markov Random Field Prior

Here, we propose Bayesian‐SBM‐MRF: the generalized version of the standard Bayesian SBM model from Section 3.1. Bayesian‐SBM‐MRF integrates two different types of taxonomic information, G and Q, as illustrated in Figure 1. We propose to replace the multinomial prior shown in Equation (2) with an MRF prior, which can incorporate information from the given taxonomic tree into microbiome community detection. The MRF [74] is a class of parametric models used for spatial data analysis that originated in theoretical physics [75, 76]. Essentially, the MRF is a nearest neighbor problem where we are interested in calculating the conditional probability that a particular taxon j belongs to community k (i.e., $z_{j}=k$) given all neighboring taxa. In this article, a neighbor is defined as any two taxa that have the same parent such as genus. Thus, the MRF prior offers a way to incorporate the taxonomic tree information Q by encouraging two taxa with the same parent to be clustered in the same community. In particular, we write that $z_{j}|z_{-j},Q∼\text{MRF}(e_{k},d)$ if the conditional probability for $z_{j}$ belonging to community k given all other taxa $z_{-j}$ and matrix Q is

(4) $$\begin{array}{ll} p(z_{j}=k|z_{-j},Q) & \propto \exp\left(e_{k}+d\underset{\left\{j^{\prime }:q_{jj^{\prime }}=1\right\}}{\sum }I(z_{j^{\prime }}=k)\right) \end{array}$$

where $d\in {\mathbb{R}}_{\geq 0}$ and $e_{k}=\log(\eta_{k})$. The MRF prior in Equation (4) reduces to the prior in Equation (2) when d=0, which means that the standard Bayesian SBM in Section 3.1 is a special case of the proposed generalized model. Additionally, the prior reduces to a non‐informative discrete uniform prior when d=0 and $e_{k}=\log(\eta_{k})=\log(1/K)$ where $\eta_{k}$ is the normalized probability of taxon j belonging to communing k given the membership of all the other taxa, and the matrices Ω and G. For that reason, we set $e_{k}=\log(1/K)$ in our model. When d>0, the available taxonomic tree information is incorporated into the model from Q. When d is too large, then $p(z_{j}=k|z_{-j})\to \infty$ and the model undergoes phase transition. A phase transition in a statistical model is concerned with how a small change in a model hyperparameter (e.g., d) can change the state or quality of overall model performance. In our simulation in Section 5.2, we determined that d=1 is a reasonable value for the MRF prior setting. Then, the full conditional posterior density for the community labels from Equation (3) is updated as

(5) $$\begin{array}{ll} & p(z_{j}=k|z_{-j},\Omega ,G,Q)\\ & \;\propto f(G|z,\Omega )p(z_{j}=k|z_{-j},Q)\\ & \;\propto \underset{k\leq k\prime }{\prod }\omega_{kk^{\prime }}^{M_{kk^{\prime }}}{\left(1-\omega_{kk^{\prime }}\right)}^{N_{kk^{\prime }}-M_{kk^{\prime }}}\exp\left(d\underset{\left\{j^{\prime }:q_{jj^{\prime }}=1\right\}}{\sum }I(z_{j^{\prime }}=k)\right) \end{array}$$

Let $\tilde{\xi }={\left({\tilde{\xi }}_{1},\ldots ,{\tilde{\xi }}_{K}\right)}^{⊤}$ and $\xi ={\left(\xi_{1},\ldots ,\xi_{K}\right)}^{⊤}$ denote the unnormalized and normalized posterior probability vectors for community membership, respectively, where ${\tilde{\xi }}_{k}=p(z_{j}=k|z_{-j},\Omega ,G,Q)$ from Equation (5) and $\xi =\tilde{\xi }/\sum_{k=1}^{K}{\tilde{\xi }}_{k}$. Then, we can sample each community membership label, $z_{j}$, from a multinomial distribution

$$z_{j}|z_{-j},\Omega ,G,Q∼\text{Mult}(1,\xi ).$$

We follow the standard Bayesian SBM to update the edge probabilities, $\omega_{kk^{\prime }}|z,G∼\text{Beta}(M_{kk^{\prime }}+a_{\omega },N_{kk^{\prime }}-M_{kk^{\prime }}+b_{\omega })$, because they are conditionally independent of the community labels z and of the taxonomic tree information Q.

## 4 Model Fitting

In this section, we first describe the details of the MCMC algorithm that employs a two‐step Gibbs sampler [77]. Then, we give the details of posterior inference for the parameters of main interest, Ω and z, as well as how to select the number of communities K.

Posterior sampling can be easily implemented for Bayesian‐SBM‐MRF since the full conditionals of each $z_{j}$ and $\omega_{kk^{\prime }}$ are multinomial and beta distributions, respectively. Algorithm 1 illustrates the model fitting steps for Bayesian‐SBM‐MRF where parameters z and Ω are jointly inferred using a two‐step Gibbs sampler. The Gibbs sampler will be run for a total of T iterations with t being the index of the current iteration. The parameter vector z can be initialized before running the Gibbs sampler by randomly assigning each $z_{j}$ a value from 1 to K from a discrete uniform probability distribution. The initialized vector is denoted as $z^{\left(0\right)}$. Each $z_{j}$ is sampled one at a time at iteration t, which is denoted as $z_{j}^{\left(t\right)}$. In general, we must condition on $z_{j}^{\left(t\right)},z_{-j},\Omega^{\left(t\right)},G,\;\text{and}\;Q$. Specifically, $z_{-j}={\left(z_{1}^{\left(t\right)},\ldots ,z_{j-1}^{\left(t\right)},z_{j+1}^{\left(t-1\right)},\ldots ,z_{p}^{\left(t-1\right)}\right)}^{⊤}$ holds for j=2,…,p−1. If j=1 or j=p, then $z_{-j}$ is expressed as either $z_{-1}={\left(z_{2}^{\left(t-1\right)},\ldots ,z_{p}^{\left(t-1\right)}\right)}^{⊤}$ or $z_{-p}={\left(z_{1}^{\left(t\right)},\ldots ,z_{p-1}^{\left(t\right)}\right)}^{⊤}$, respectively.

ALGORITHM 1 **Gibbs sampler for Bayesian‐SBM‐MRF. Note that $z_{-j}={\left(z_{1}^{\left(t\right)},\ldots ,z_{j-1}^{\left(t\right)},z_{j+1}^{\left(t-1\right)},\ldots ,z_{p}^{\left(t-1\right)}\right)}^{⊤}$ holds for j=2,…,p−1; $z_{-j}={\left(z_{2}^{\left(t-1\right)},\ldots ,z_{p}^{\left(t-1\right)}\right)}^{⊤}$ holds for j=1, and $z_{-j}={\left(z_{1}^{\left(t\right)},\ldots ,z_{p-1}^{\left(t\right)}\right)}^{⊤}$ holds for j=p. The vectors $\tilde{\xi }={\left({\tilde{\xi }}_{1},\ldots ,{\tilde{\xi }}_{K}\right)}^{⊤}$ and $\xi ={\left(\xi_{1},\ldots ,\xi_{K}\right)}^{⊤}$ contain the unnormalized and normalized probabilities, respectively.**

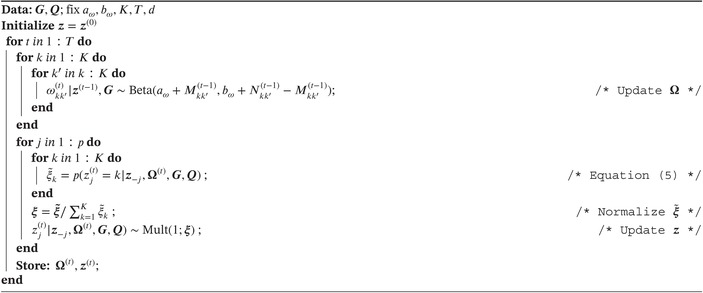

From the posterior samples obtained *via* Algorithm 1, we make three inferences that will provide a comprehensive scope of the community structure of the microbiome co‐occurrence network. The first is to infer the edge probabilities in Ω so that we can assess the relationship of all taxa within and between the communities. The second is to identify the community labels of the p taxa, which are collected in the parameter vector z. Thirdly, we would like to infer the optimal value of K to estimate the appropriate number of communities.

Bayesian inference commonly uses simple numerical summaries such as the posterior mean to obtain a point estimate of a model parameter [73]. The point estimate for each edge probability $\omega_{kk^{\prime }}\in \Omega$ is computed as the posterior mean of the after burn‐in posterior samples, which is given by

$${\hat{\omega }}_{kk^{\prime }}=\frac{1}{T-B}\overset{T}{\underset{t=B+1}{\sum }}\omega_{kk^{\prime }}^{\left(t\right)}$$

where t is the current iteration of the MCMC algorithm after a burn‐in period of B iterations, and T is the total number of iterations.

Next, we also provide a posterior point estimate of the community labels of all p taxa *via* $z^{\left(t\right)}$ at a particular iteration t. We could identify the $z^{\left(t\right)}$ at a particular iteration t (after burn‐in) that maximizes the posterior distribution. This is known as the *maximum a posteriori* (MAP) estimate and is denoted as ${\hat{z}}^{\text{MAP}}$. Specifically,

$${\hat{z}}^{\text{MAP}}=\underset{t\in \{B+1,\ldots ,T\}}{\text{argmax}}p(z^{\left(t\right)}|\Omega^{\left(t\right)},G,Q).$$

To avoid the issue of label switching in z at each iteration of the MCMC algorithm, we first arranged the intra‐block edge probabilities (i.e., each $\omega_{kk^{\prime }}$ where $k=k^{\prime }$) in order from greatest to least. Then, the community with the greatest intra‐block edge probability is relabeled as community 1, the community with the second greatest intra‐block edge probability is relabeled as community 2, and so forth.

Bayesian information criterion (BIC) [78] is a popular metric for estimating the optimal number of communities, K, for model‐based clustering algorithms [79, 80]. BIC is defined as $\text{BIC}=\nu \log p-2\log p(G,Q|\hat{z},\hat{\Omega })$ where ν is the number of model parameters and p is the number of taxa. Here, the number of model parameters is ν=p+K(K+1)/2 since we are estimating p community membership labels for the parameter z and K(K+1)/2 edge probabilities for the parameter Ω.

## 5 Results

In Section 5.1, we provide the results of our model on a real urinary microbiome dataset. To the best of our knowledge, this is the first time anyone has studied the network and community structure of the urinary microbiome with respect to recurrent urinary tract infections (rUTI) in postmenopausal women. In Section 5.2, we also validate Bayesian‐SBM‐MRF by assessing its community detection performance across various simulation settings.

### 5.1 Real Data Analysis: Urinary Microbiome Data

We applied Bayesian‐SBM‐MRF to the urinary microbiome data from our study on rUTI with n=75 postmenopausal female patients. Our findings below are important for translational medicine because they provide a new foundation for future studies on urinary microbiome community structure. Knowledge of this niche and the role of the urinary microbiota in susceptibility to urinary tract infections (UTI) is only in its infancy. For example, UTIs are currently understudied even though they are the most common of adult infections and postmenopausal women are disproportionately affected by rUTI [81]. Ammitzbøll et al. [82] reported that premenopausal and postmenopausal women displayed different core urinary microbiota at the genus level, providing strong rationale to characterize the postmenopausal urinary microbiome in urinary disease.

The urinary microbiome data originally had 180 bacterial species along with their known genera. We filtered out all species that had fewer than seven non‐zero counts, which resulted in a total of p=99 species from 41 genera. Of the 41 genera, 18 of these had at least two species belonging to the same genus. About 77% of the bacterial species belong to these 18 genera. Each of the remaining 23% of species belongs to unique genera. The full details regarding the study, data, and metagenomic sequencing can be found in Neugent et al. [81] The microbiome co‐occurrence network G was estimated from the species‐level abundance data and the available taxonomic tree information was incorporated into Q as described in Section 2. The MCLR transformation was done using the MCLR function from the SPRING library in R. Then, we performed community detection on G using Bayesian‐SBM‐MRF with d=1 for the MRF prior setting. We set $\eta_{k}=\log(1/K)$ to impose a non‐informative discrete uniform prior on each community label $z_{j}$. We set $a_{\omega }=b_{\omega }=1$ to impose a non‐informative uniform prior on each edge probability $\omega_{kk^{\prime }}$. We ran T=1000 iterations, with the first half of the iterations discarded as burn‐in samples.

Using BIC, we determined that K=7 was the optimal number of communities for these data. While selection of elbows in a scree plot can be arbitrary, it was our opinion that the first useful elbow was at K=7 (see the scree plot in Figure S1 in the Supporting Information). Despite an earlier elbow at K=2, we decided not to set K=2 since the resulting partition would be excessively coarsened for the purpose of this application. In order to assess model convergence, we ran four parallel chains of the MCMC algorithm with different starting points (i.e., z in each chain was initialized at random as described in Section 4). Then, we calculated the ARI between the chains. ARI varied between 0.88 and 0.95 with a mean of about 0.91, indicating that the results (i.e., each $\hat{z}$) were very similar. Thus, there were no concerns about convergence issues.

**FIGURE 2:**
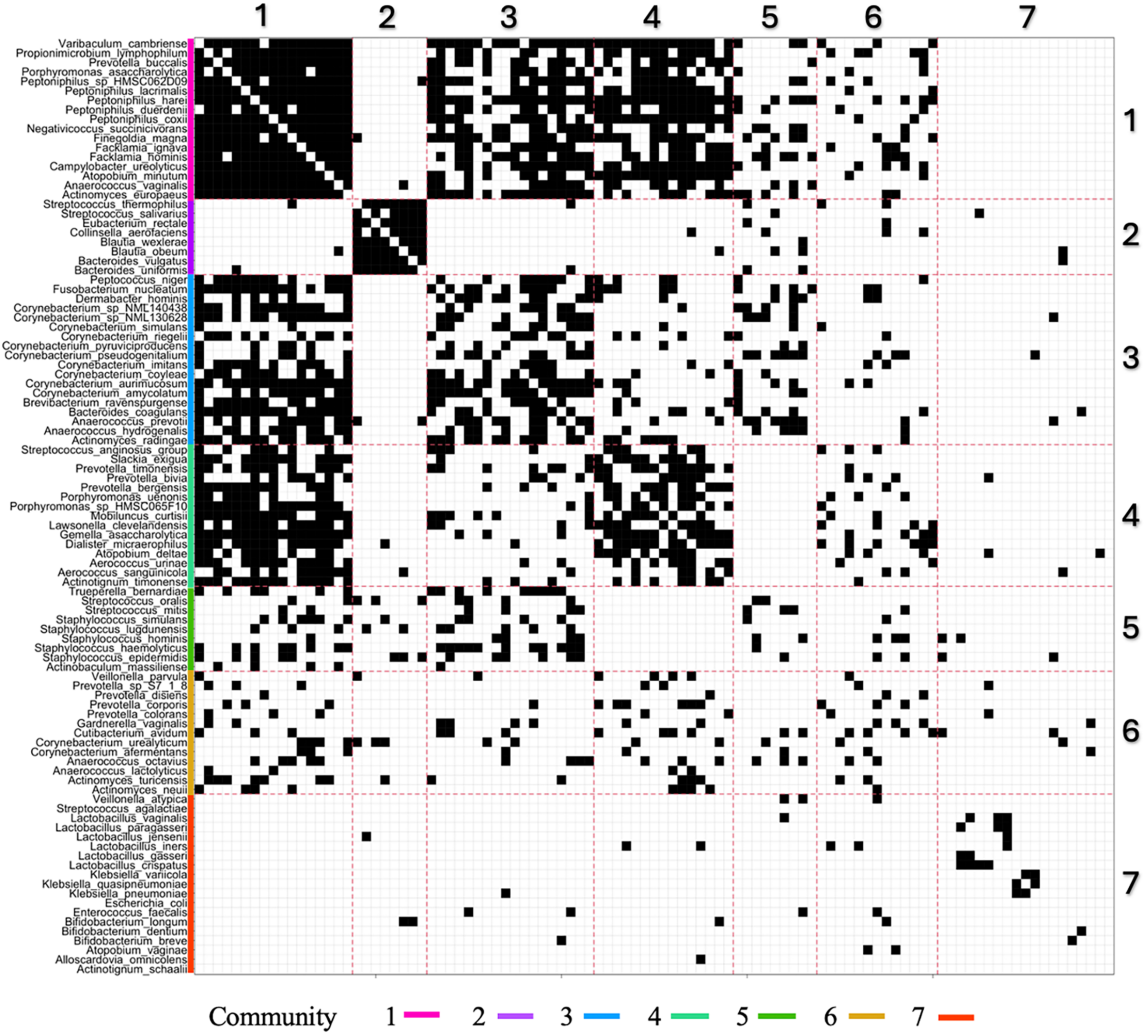
A 99×99 heatmap of K=7 communities estimated by Bayesian‐SBM‐MRF (d=1). A black pixel indicates a significant association (edge) between two taxa and a white pixel indicates no association (no edge). The considered 99 species are grouped in the margin by community. Communities are identified by color and number in the margin, which are the same identifiers used in Figure 3. The red dashed lines partition the heatmap into seven rows and columns of blocks indicated at the top and right side of the plot. [Colour figure can be viewed at wileyonlinelibrary.com]

**FIGURE 3:**
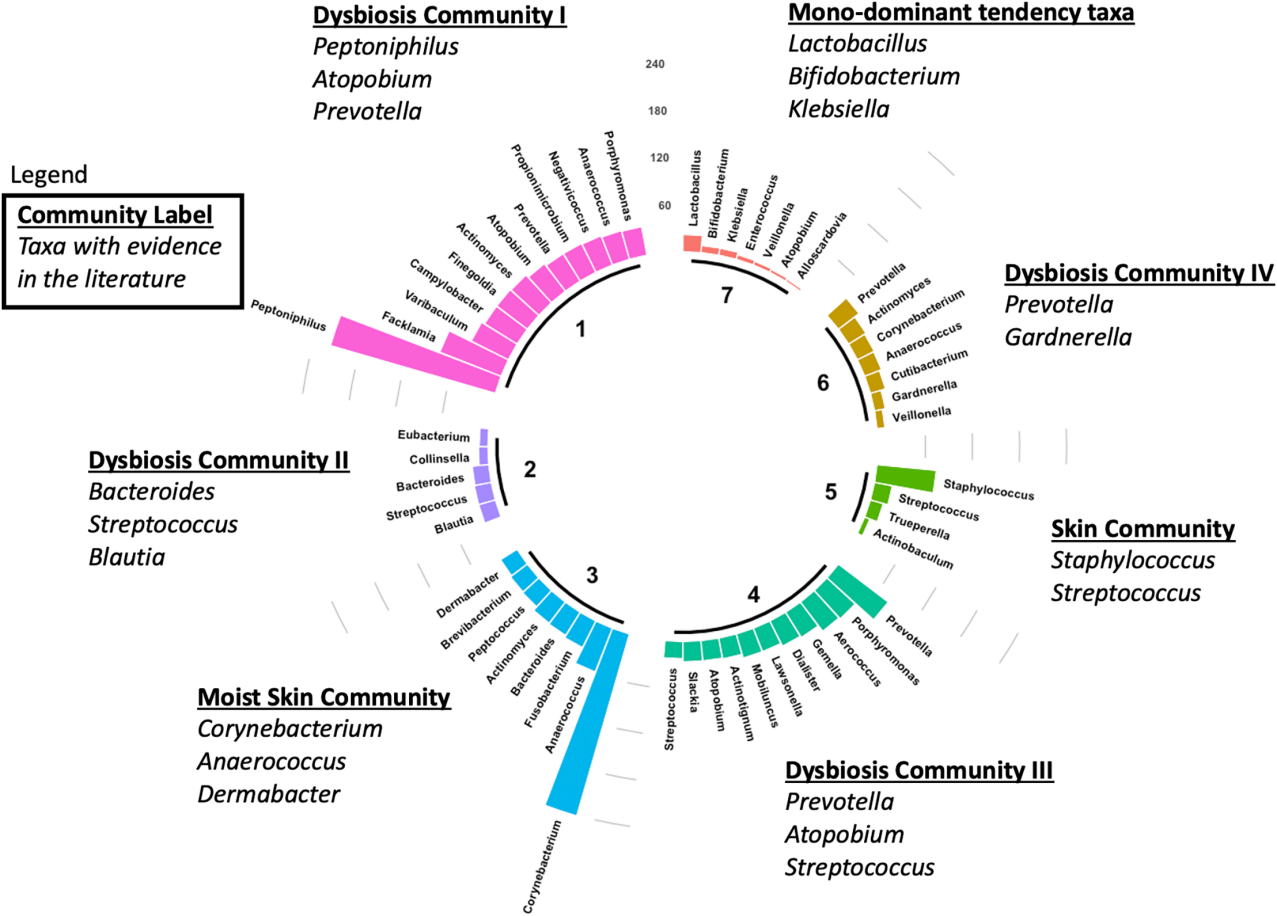
A circular bar plot of intra‐network nodal degree grouped by genus for the K=7 communities detected by Bayesian‐SBM‐MRF (d=1). Labels on the bars identify the genera found in each community. Annotations identify genera with evidence‐based characteristics. [Colour figure can be viewed at wileyonlinelibrary.com]

The 99×99 heatmap in Figure 2 helps to visualize and organize the nodes and edges of the urinary microbiome co‐occurrence network G into the seven communities estimated by our model. Names of genera and species are in the margin with their community label indicated by both a color and number. The same community identifiers are also used in Figure 3. Black and white pixels represent edges and non‐edges, respectively, between any two taxa. An edge represents a significant association between the two corresponding taxa. The red dashed lines partition the heatmap into seven rows and columns of blocks where block $\left(k,k^{\prime }\right)$ indicates the block in row k and column $k^{\prime }$. The labels 1,…,7 for k and $k^{\prime }$ are listed at the top and right side of the plot. Blocks on the main diagonal where $k=k^{\prime }$ represent intra‐community associations. Off‐diagonal blocks where $k\neq k^{\prime }$ represent inter‐community associations. The grid is symmetric about the main diagonal, so block $\left(k,k^{\prime }\right)$ is the transpose of block $\left(k^{\prime },k\right)$. The main diagonal blocks, which are positioned from top‐left to bottom‐right, represent the edges within the seven communities identified by the model. Such communities are arranged in order starting at top from greatest to least intra‐block edge probability (a higher density of black pixels indicates a greater probability of an edge between any two nodes within a block). Thus, community 1 has the greatest intra‐block edge probability as visible in block (1,1); community 2 has the second greatest intra‐block edge probability, and so on. Interestingly, the taxa that co‐occur in communities 1 and 2 have been observed before as co‐occurring in two separate and diverse communities in a previous work of ours [81]. Also, notice that the sizes of the seven communities are quite different *a posteriori* even though we imposed a discrete uniform prior on the community labels. This is likely the result of the model learning the communities. Next, black pixels in off‐diagonal blocks represent inter‐block edges connecting taxa in two different blocks. For example, the black pixels in block (1,3) illustrate edges connecting the taxa in communities 1 and 3. Using symmetry, one could also refer to block (3,1) as well. Communities 1 and 2 have similar intra‐block pixel density, which indicates that they have similar intra‐block edge probabilities. Even though they are similar in that sense, the model separates them because their associations with other communities are different. In fact, Community 1 has more associations with Communities 3 and 4; whereas, Community 2 barely associates with any other community (many off‐diagonal white pixels). More associations between community 1 and other communities may also indicate that taxa within this community have more synergistic metabolic interactions with other taxa both within its own community and with other communities. On the other hand, community 2 species may interact less with taxa in other communities because they are more metabolically independent.

We calculated the nodal degree of the genera in each community as a way to explore the biological relevance of genus and community. Nodal degree in an undirected binary network is the number of edges between a particular genus in a community and all other genera within that community or the network. Genera with higher intra‐community or intra‐network nodal degree have more and stronger connectivity making them central members within their community or network, respectively. These are often referred to as “hub taxa” within the microbiome literature and they may possibly influence microbiome structure and function [5, 83]. The circular bar plot in Figure 3 displays the intra‐network nodal degree of genus by community (Figure S2 in the Supporting Information displays both the intra‐community and intra‐network nodal degree). The communities and the genera within them have evidence‐based characteristics that are of biological interest. These characteristics are noted in the plot annotations next to each community. For example, Communities 3 and 5 are associated with the human skin microbiome [84]. We found four distinct communities (1, 2, 4, and 6) harboring taxa known to be markers of dysbiosis in the vaginal microbiome from genera such as *Gardnerella, Prevotella, Streptococcus, Bacteroides, Blautia*, and *Peptoniphilus* [81, 85, 86]. It has been reported that the vaginal and urinary microbiomes are interconnected with one another [87, 88]. *Peptoniphilus* and *Prevotella* are hubs in communities 1 and 4, respectively, since they have the greatest nodal degrees. Interestingly, *Peptoniphilus* and *Prevotella* are hypothesized to be markers of dysbiosis in the urinary and vaginal microbiomes [81, 85, 86, 89] as well as hubs in the vaginal microbiome during dysbiosis [90, 91, 92]. These hub taxa along with other co‐occurring taxa may help to define community structure in urinary microbiomes of women with increased rUTI susceptibility [81, 87]. Future research is needed to validate signatures of urinary dysbiosis associated with rUTI susceptibility.

We also compared the results of Bayesian‐SBM‐MRF when d=0 (no taxonomic tree information is leveraged) versus d=1 (taxonomic tree information is leveraged) as a way to explore community diversity by genus. Table S3 in the Supporting Information gives some promising insight into the behavior of our model. When d=1, we found at least six genera that appear in fewer communities compared to d=0. While *Peptoniphilus*, *Facklamia*, *Bifidobacterium*, and *Staphylococcus* collapsed into one community. *Corynebacterium* collapsed from four to two communities and *Anaerococcus* collapsed from four to three communities. All other genera showed no changes in the number of assigned communities. Thus, Bayesian‐SBM‐MRF does not simply force species from the same genus into one community. These observations may indicate that Bayesian‐SBM‐MRF is well‐behaved because it is likely making a balanced use of both the microbiome co‐occurrence network G and the taxonomic tree information Q. Last, Section S2 in the Supporting Information extends our comparison with an exploratory analysis of the diversity with respect to genus within each community.

### 5.2 Simulation

#### 5.2.1 Generative Model

We simulated the microbiome co‐occurrence network G with species‐level taxa as nodes that are grouped into K communities. First, we simulated co‐occurrence networks that are composed of K=3,6,9,12 communities and p=180 species‐level taxa. We assumed an equal number of taxa per community and assigned a community label to each taxon indicated by $z_{j}$. An edge between any two taxa of the network was sampled as $g_{jj^{\prime }}|\omega_{kk^{\prime }}∼\text{Bernoulli}(\omega_{kk^{\prime }})$. Edge probabilities between taxa in separate communities are typically low, so we randomly sampled their edge probabilities from a uniform distribution such that $\omega_{kk^{\prime }}∼\text{Uniform}(0,0.1)$ for $k\neq k^{\prime }$. In contrast, the probability $\omega_{kk}$ of an edge between two taxa within the same community where $k=k^{\prime }$ took on preset values between 0 and 1 to imitate various levels of taxon‐taxon associations in each community. Specifically, we set $\omega_{kk}=0.3,0.6,0.95$ for k=1,2,3 (when K=3), $\omega_{kk}=0.1,0.3,0.5,0.7,0.9,0.97$ for k=1,…,6 (when K=6), $\omega_{kk}=0.12,0.2,0.3,0.4,0.5,0.7,0.8,0.9,0.99$ for k=1,…,9 (when K=9), and $\omega_{kk}=0.1,0.2,0.25,0.3,0.4,0.5,0.6,0.7,0.8,0.85,0.9,0.97$ for k=1,…,12 (when K=12).

Next, we generated matrix Q to incorporate an additional level of taxonomic tree information. Let $\tau ={\left(\tau_{1},\ldots ,\tau_{p}\right)}^{⊤}$ denote the genus labels for species 1,…,p. To incorporate the taxonomic tree information, we randomly assigned each of the p species to a genus $\tau_{p}$ and community k using three strength settings (weak, moderate, and strong). Strength here describes how informative one additional level of the taxonomic tree (i.e., genus) is for community assignment. To quantify strength, we used the adjusted Rand index (ARI) [93], which is a similarity metric between two sets of discrete labels (e.g., community versus genus; or estimated clusters versus true clusters). The ARI of two sets of discrete labels z and $\hat{z}$ is computed as

ARI(z,z^)=p2(a+d)−[(a+b)(a+c)+(c+d)(b+d)]p22−[(a+b)(a+c)+(c+d)(b+d)]

where $a=\sum_{j>j^{\prime }}I(z_{j}=z_{j^{\prime }})I({\hat{z}}_{j}={\hat{z}}_{j^{\prime }})$, $b=\sum_{j>j^{\prime }}I(z_{j}=z_{j^{\prime }})I({\hat{z}}_{j}\neq {\hat{z}}_{j^{\prime }})$, $c=\sum_{j>j^{\prime }}I(z_{j}\neq z_{j^{\prime }})I({\hat{z}}_{j}={\hat{z}}_{j^{\prime }})$ and $d=\sum_{j>j^{\prime }}I(z_{j}\neq z_{j^{\prime }})I({\hat{z}}_{j}\neq {\hat{z}}_{j^{\prime }})$. ARI has an expected value of zero when computed between independent random labelings, and a maximum value of one when computed between labelings corresponding to the same partition. ARI can be negative (minimum ARI is −1) when the clustering result is worse than what would be expected by random chance [93, 94].

**FIGURE 4:**
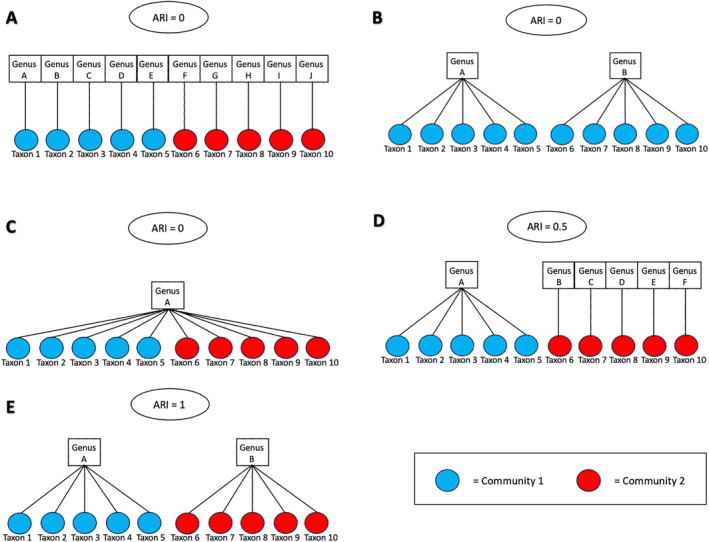
Illustrative examples of weak (A, B, C), moderate (D), and strong (E) association between genus and community with ten taxa, their genera, and two communities. Strength is quantified by the ARI between genus and community labels. Genera are represented by squares, taxa by circles, and community by circle color (red, blue). [Colour figure can be viewed at wileyonlinelibrary.com]

Here, we use ARI to categorize the relationship between genus and community labels. We considered the taxonomic tree information to be weakly informative if the ARI between the genus and community labels was low (e.g., ARI(z,τ)≤0.3), moderately informative if 0.3<ARI(z,τ)≤0.7 and strongly informative if 0.7<ARI(z,τ)≤1. Figure 4 provides illustrative examples of the weak, moderate, and strong simulation settings. There are at least three possible scenarios under the weak setting where the ARI of genus and community is zero: examples A, B, and C. None of the taxa in the same community belong to the same genus (example A); or all the taxa and genera collapse into one community (example B); or taxa of the same genus are grouped into two different communities rather than together (example C). The moderate setting where ARI≈0.5 (example D) exhibits some similarity between the genus and community: the taxa from the first community all belong to the same genus, while the taxa in the second community all belong to distinct genera. The strong setting where ARI=1 (example E) illustrates the perfect scenario of maximum similarity where taxa belonging to the first community all belong to the same genus, and taxa in the second community all belong to another genus.

We included 30 genera in total in the simulated data. There were 12 simulation settings, resulting from combinations of 3 levels of association between genus and community and 4 values of K. For each scenario, we repeated the above steps to generate 50 replicates.

#### 5.2.2 Evaluation Metrics

First, we wanted to determine if Bayesian‐SBM‐MRF improves the performance of the standard Bayesian SBM by incorporating taxonomic tree information. We conducted a sensitivity analysis to compare the results of Bayesian‐SBM‐MRF under four settings of the MRF prior when d=0,0.5,1,2. When d=0, the standard Bayesian SBM is employed since no taxonomic tree information is incorporated. This setting was then compared to the other three when d>0 so that Bayesian‐SBM‐MRF incorporates taxonomic tree information. Specifically, this part is a sensitivity analysis that can help to determine a reliable value for d. Second, we compared Bayesian‐SBM‐MRF to several competing models that are listed in Table S1. The competitors we considered are ESBM [38], sbm‐cov [35], the Louvain algorithm [95], cluster_fast_greedy function [96] found in the very popular igraph package in R [97], and spectral clustering from the anocva package in R [40]. ESBM and sbm‐cov are Bayesian and frequentist model‐based approaches, respectively, that can leverage nodal covariates. For these two methods, we provided the genus labels as categorical nodal covariates. It is worth noting that neither ESBM nor sbm‐cov were applied to microbiome network data. ESBM offers parametric and nonparametric Bayesian SBMs with several choices of priors such as Dirichlet‐multinomial (DM) and Dirichlet process (DP) priors, respectively. ESBM (DM) and ESBM (DP) are finite and infinite mixture models, respectively. The other methods are algorithmic‐based clustering approaches and are not able to leverage nodal covariates. We used the recommended or default model settings specified by the authors of each competing method.

We compared the performance of all models on the 50 replicated data sets under each setting to determine how well they can recover the true community labels z using ARI. Specifically, ARI is a commonly used validation metric for clustering methods to determine how well they can recover the true clusters [93, 98, 99, 100, 101, 102, 103, 104, 105]. An ARI closer to one indicates stronger similarity between z and $\hat{z}$; whereas, an ARI closer to zero indicates that their similarity is no better than independent random labelings. Further, ARI is the corrected‐for‐chance version of the Rand index [106] so that the common issue of label switching by clustering algorithms is not problematic when comparing estimated and true community labels. For example, an algorithm may estimate the community labels of four taxa to be $\hat{z}={\left(1,1,2,2\right)}^{⊤}$ while the underlying truth may be $z={\left(2,2,1,1\right)}^{⊤}$. Both sets of labels are equivalent because the first two taxa are assigned to the same community and the other two taxa are assigned to the other community (in fact, ARI$(z,\hat{z})=1$ for this example). ARI as a validation measure for clustering methods is better than other validation metrics such as the area under the receiver operating characteristic curve (AUC), precision, recall, accuracy, and so forth, because these metrics do not account for label switching [98]. In the above example, the accuracy of z with respect to $\hat{z}$ is zero due to label switching. This illustrates why accuracy is not a useful metric for validating a clustering model.

#### 5.2.3 Simulation Results

**FIGURE 5:**
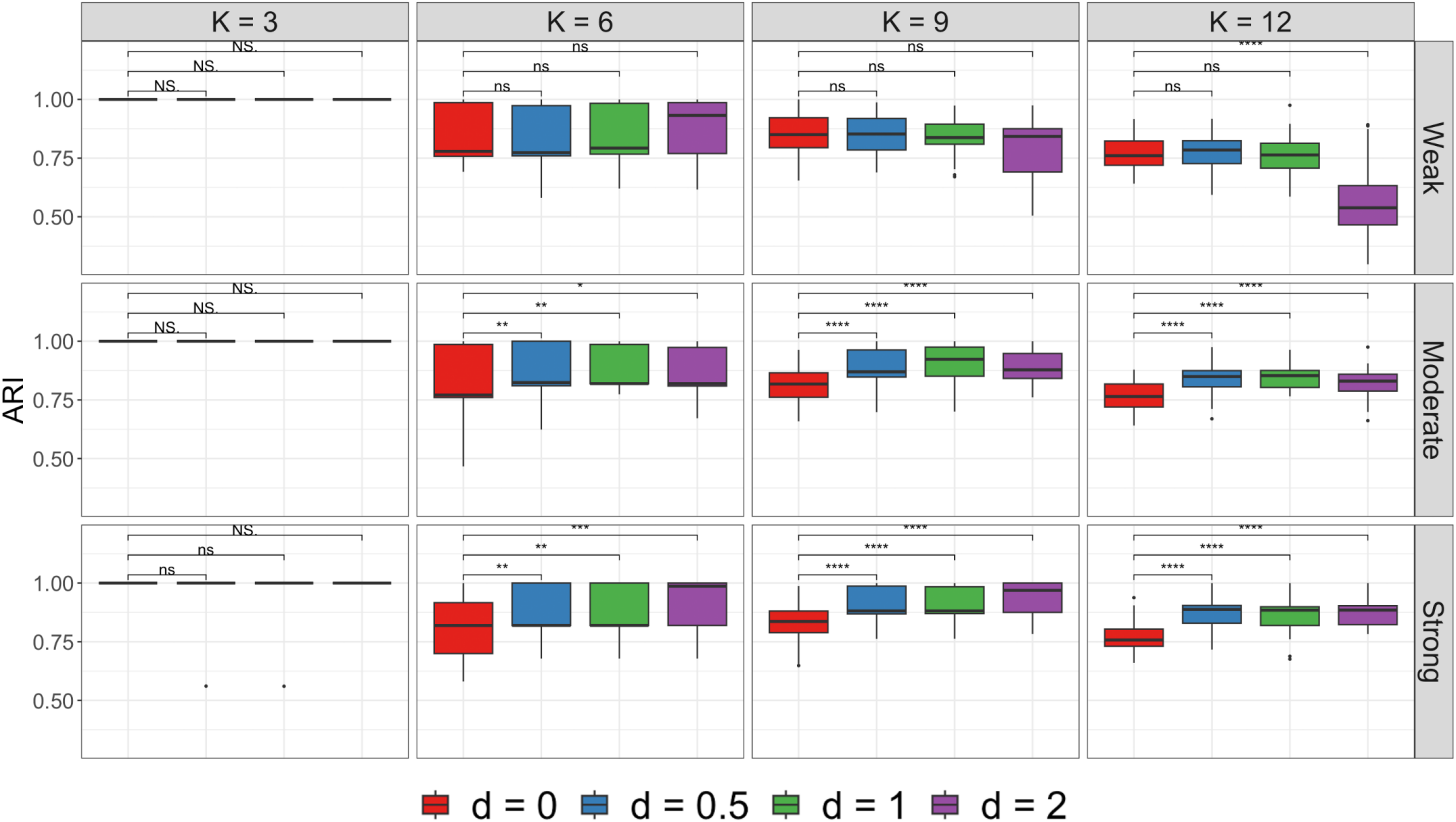
Boxplots of the ARI between the true and estimated partitions with four settings of the MRF prior (d=0,0.5,1,2) for model sensitivity analysis using the simulated data sets of communities with size K=3,6,9,12 and weak, moderate, and strong levels of informative strength of taxonomic tree information. The paired Wilcoxon test was performed to compare the standard SBM where d=0 to the other settings where d>0 to assess model performance without and with taxonomic tree information. Significance is indicated by *(p<0.05), **(p<0.01), ***(p<0.001), ****(p<0.0001), ns (not significant with p∈(0.05,1)), NS (not significant with p=1). [Colour figure can be viewed at wileyonlinelibrary.com]

First, we conducted a sensitivity analysis on our model to determine an appropriate d value for the MRF prior. As d increases, the MRF prior increases the influence of the taxonomic tree information. However, too much influence may result in poor performance as described earlier regarding phase transition. Results of the sensitivity analysis are displayed in Figure 5. The ARI between the true community labels z and the estimated community labels $\hat{z}$ was computed using the ARI function from the aricode library in R. We performed the paired Wilcoxon test to compare the standard Bayesian SBM when d=0 to the other three settings of our generalized model with d=0.5,1,2. Several important observations stand out about our generalized SBM model. The first is that when the level of strength of the taxonomic tree information is moderate or strong, Bayesian‐SBM‐MRF where d>0 has significantly higher ARI than d=0 (apart from the case when K=3). This indicates that the taxonomic tree information can be useful for recovering the true community labels, especially when such a tree is informative. Second, there are no significant differences in ARI for d=0 versus d=0.5,1 when strength is weak, which indicates that a modest inclusion of taxonomic tree information does not hurt the performance of our generalized model even when such a tree is not informative. Additionally, the difference between d=0 and d>0 is never significant when K=3, irrespective of the strength of taxonomic tree information. This is arguably due to the fact that fewer blocks reduce the complexity of the clustering task. Thus, different values for d will likely result in similar model performance when complexity is lower (i.e., when K=3). When K=9 or 12 and strength is weak, the boxplot corresponding to d=2 exhibits a decline in ARI indicating that too much inclusion of taxonomic tree information hurts model performance. We can probably expect that such a decline may become even more significant for larger d values, hence we avoid using these values. In conclusion, we recommend using d=1 for Bayesian‐SBM‐MRF because our model performs equally or better than the other settings under all scenarios.

**FIGURE 6:**
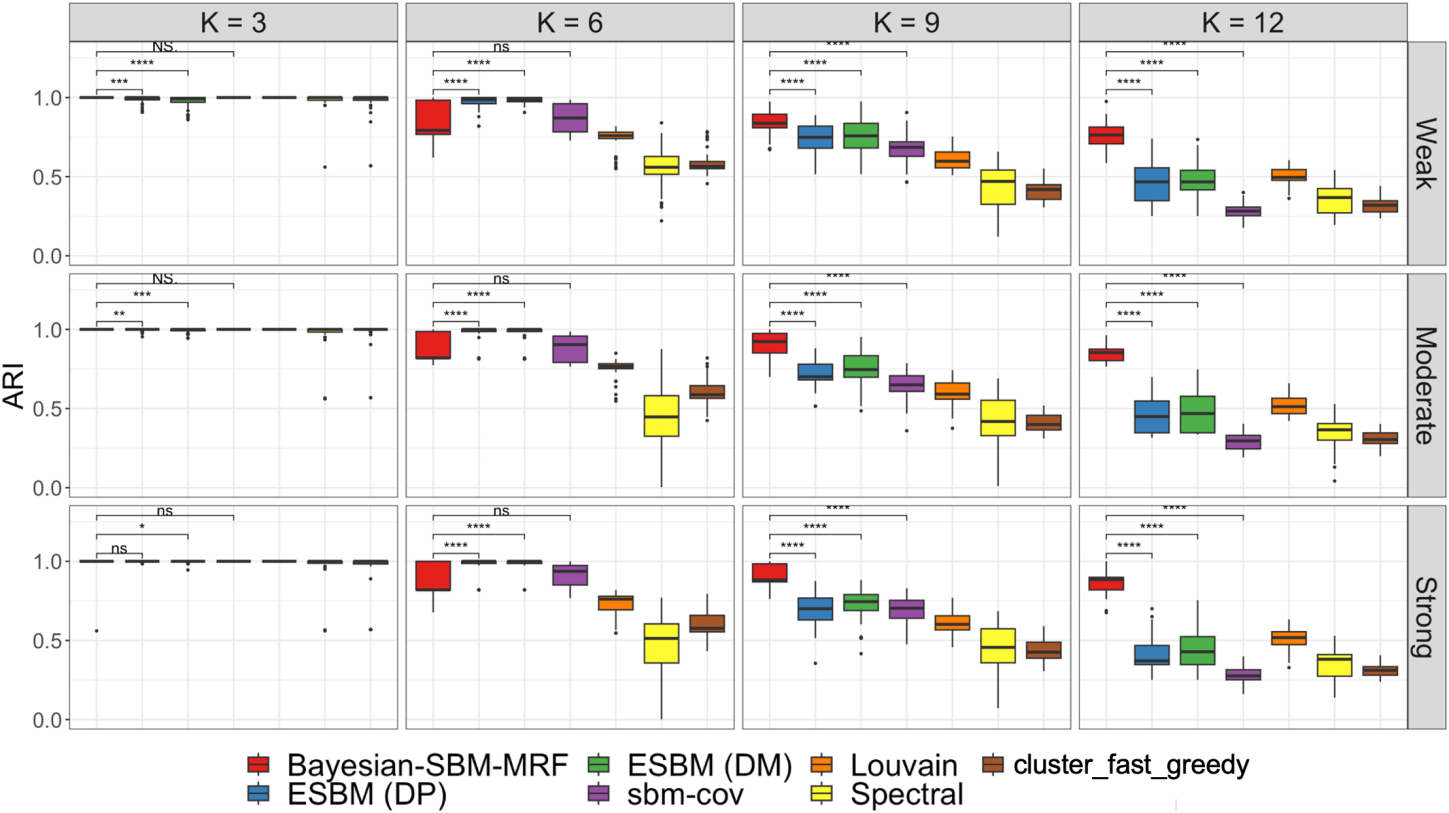
Boxplots of the ARI between the true and estimated partitions of competing methods along with Bayesian‐SBM‐MRF when d=1 for the simulated data sets of communities with size K=3,6,9,12 and weak, moderate, and strong levels of informative strength of taxonomic tree information. The paired Wilcoxon test was performed to compare our model to those that are able to incorporate nodal covariates. Significance indicators are the same as described in Figure 5. [Colour figure can be viewed at wileyonlinelibrary.com]

Next, we compared our model with d=1 to the competitors listed in Section 5.2.2. The boxplots of ARI between z and $\hat{z}$ for all competing models in Figure 6 demonstrate that Bayesian‐SBM‐MRF has superior performance under most simulation settings especially as K increases. When K=3, all models perform well since most ARI distributions concentrate close to one. While ARI decreases slightly for our model as K increases, the ARI of all competitors suffer a much greater decrease. The competitors that do not leverage nodal covariates (i.e., Louvain, Spectral, and cluster_fast_greedy) have the lowest ARI compared to the others with the exception of the Louvain algorithm when K=12. We used the paired Wilcoxon test to compare our model to the methods that can leverage nodal covariates (i.e., ESBM (DM), ESBM (DP), and sbm‐cov) whose performance was closest to our method. Compared to sbm‐cov, our model performed as good (when K=3,6) or significantly better (when K=9,12). Compared to ESBM, our model performed equally good for K=3, significantly worse for K=6, and significantly better for K=9,12.

Finally, note that ARI was used in two unrelated ways in this simulation study. First, we used ARI to categorize the relationship strength between one part of taxonomic tree (i.e., genus) and community labels given by z as weak, moderate, or strong. This was one step of the generative process in Section 5.2.1 that generated Q. The generation of the microbiome co‐occurrence network G was a separate step not captured by this use of ARI. Second, ARI was used to validate our model in Section 5.2.3. Here, we calculated the ARI between z and $\hat{z}$, which captured the similarity between the true and predicted community labels.

## 6 Discussion

In this article, we develop a two‐stage method for community detection on a microbiome co‐occurrence network that accounts for the challenging characteristics of microbiome data, leverages taxonomic tree information *via* the novel use of the MRF prior, outperformed competing methods in our simulation, and produced biologically meaningful results in our novel real data analysis of the urinary microbiome. The first stage estimates the microbiome co‐occurrence network from the MCLR‐transformed relative abundances, which is an important step that accounts for their compositionality, non‐linearity, and zero‐inflation. To the best of our knowledge, we believe that this is the first time the MCLR transformation has been used on microbiome data for community detection. The second stage takes the estimated microbiome co‐occurrence network and available taxonomic tree information into account to perform community detection using our generalized SBM model known as Bayesian‐SBM‐MRF. We believe that Bayesian‐SBM‐MRF is the first of its kind to include two levels of the taxonomic tree using a MRF prior, which makes our model an attractive tool for community detection. The simulation study revealed several advantages of Bayesian‐SBM‐MRF. First, the inclusion of taxonomic tree information improves model performance when such a tree is informative. Second, a moderate inclusion of taxonomic tree information does not hurt model performance even when such a tree is non‐informative. Third, Bayesian‐SBM‐MRF demonstrated superior performance in most simulation settings when compared to competing methods regardless of their ability to leverage nodal covariates. When applied to real microbiome data, our model identified distinct communities of taxa with evidence‐based characteristics that are biologically meaningful. Hence, Bayesian‐SBM‐MRF provides a new tool for facilitating advanced microbiome studies.

There are several limitations of Bayesian‐SBM‐MRF. First, we account for zero‐inflation in the abundance data using an external normalization step (i.e., the MCLR transformation). Other methods, such as HARMONIES [12] and SPRING [58], proposed zero‐inflated models for estimating a microbiome network using probability distributions such as the zero‐inflated negative binomial distribution. Both methods used model‐based or internal normalization and demonstrated superior performance over well‐known network analysis methods. Second, while BIC is standard and widely used for selecting the optimal K, some issues exist. First, the choice of K from a BIC elbow plot is subjective. Second, alternative selection criteria have been proposed that make certain claims about BIC. Recently, Hu et al. [107] proposed a corrected BIC (CBIC) designed specifically for SBMs to find the optimal K by imposing an additional penalty on the log‐likelihood. They found that standard BIC may overestimate the true number of communities. Biernacki et al. [108] proposed the integrated complete likelihood (ICL) for mixture models and also found that BIC may overestimate K. Third, our model assumes a fixed K. Consequently, it does not account for uncertainty on K, and must be run for multiple values of K until the optimal value of K is determined *via* BIC. Whereas, nonparametric Bayesian models with random or infinite K (as the sample size diverges) do not require multiple runs to determine the optimal number of clusters, and they allow to quantify uncertainty on it [20, 38, 109]. Despite such limitations, our model still exhibits excellent performance overall, arguably due to inclusion of the MRF prior. Each of these limitations can be explored in future work to possibly help improving model performance.

Lastly, the findings of our real data analysis are important for translational medicine because they provide a new foundation for future studies on the urinary microbiome and human health. To the best of our knowledge, this was the first time community detection has ever been done to study the structure of the urinary microbiome with respect to rUTI from postmenopausal women. While Bayesian‐SBM‐MRF detected distinct communities with taxa known to be associated with dysbiosis in the vaginal microbiome, additional studies will be required to validate the biological relevance of these communities in the urinary microbiome and their association with rUTI. In fact, our knowledge of the urinary microbiome is in its infancy. For example, we do not have a clear picture which taxa represent true commensals and which are dysbiotic or possibly pathogenic. This lack of understanding makes sequencing‐based diagnostic methods difficult to implement clinically as we lack definitive microbial biomarkers of health and disease beyond those that are easily cultivable for several urologic conditions. By uncovering community structure within the urinary microbiome, taxa with heretofore unknown functions can be classified into community types defining different disease states elucidating their potential function in the urinary microbiome or association with a disease state. In conclusion, our model provides a new powerful tool for uncovering community structure in microbiome co‐occurrence networks.

## Conflicts of Interest

The authors declare no conflicts of interest.

## Supporting information




**Data S1** Supporting Information.

## Data Availability

Whole genome metagenomic sequencing read data (FASTQ files) have been deposited onto the NIH Sequence Read Archive (SRA) under the BioProject number [NCBI]: PRJNA801448. Prior to depositing, all human‐mapping reads were removed from the data. Simulated data, filtered real urinary microbiome abundance data used for analysis, and the related source code in R are available at https://github.com/klutz920/Bayesian‐SBM‐MRF.
