# Supplementary material for: A Generalized Bayesian Stochastic Block Model for Microbiome Community Detection
Source: Stat Med. 2025 Jan 24;44(3-4):e10291. doi: 10.1002/sim.10291 (PMC11760646; doi:10.1002/sim.10291)
Supplement: Supplementary file 1 — Data S1 Supporting Information. [file SIM-44-0-s001.zip › supplement.pdf]

S1 — SUPPLEMENTAL TABLES AND FIGURES

Supplemental Materials for
"A generalized Bayesian stochastic block model for microbiome community detection"

TABLE S1 Available software for stochastic block models in various programming languages and whether or not they can leverage covariates (Yes or No). Abbreviations: (i) Approach is either Bayesian (Bayes), frequentist (Freq), or algorithmic (Alg); (ii) Graph is either directed (D) or undirected (U); (iii) Data can be binary (B), discrete (P), or continuous (C); (iv) Companion publications for some of the packages are currently not available (NA).

Package	Approach	Graph	Data	Covariates	Language	Repository	Publication
anocva	Alg	U	B,P,C	No	R	CRAN	Vidal et al [1]
BipartiteSBM	Bayes	U	B	No	Python/C++	GitHub	NA
blockmodeling	Alg	U	B,P,C	No	R/C/Fortran	CRAN	Žiberna et al [2]
blockmodels	Freq	D,U	B,P,C	Yes	R	CRAN	Leger et al [3]
CommunityDetection	Alg	U	B	No	Python	GitHub	NA
dBlockmodeling	Alg	D,U	B,P,C	No	R	CRAN	Brusco et al [4]
ESBM	Bayes	U	B	Yes	R	GitHub	Legramanti et al [5]
expSBM	Freq	D,U	B,C	No	R	CRAN	Rastelli et al [6]
graphon	Bayes	U	B	No	R	CRAN	Orbanz et al [7]
graph-tool	Bayes	D,U	B,P,C	Yes	Python	Online	Peixoto et al [8]
greed	Bayes	D,U	B,P,C	No	R	CRAN	Cǒme et al [9]
GREMLIN	Freq	D,U	B,P,C	No	R	CRAN	Bar-Hen et al [10]
igraph	Alg	D,U	B,P,C	No	R	CRAN	Csardi et al [12]
I-Louvain	Alg	U	C	Yes	Python	Dropbox	Combe et al [13]
missSBM	Freq	D,U	B	Yes	R	CRAN	Barbillon et al [14]
MODE-NET	Alg	U	B	No	C++	Online	NA
noisySBM	Freq	D,U	C	No	R	CRAN	Rebafka et al [15]
pysbm	Bayes, Freq	D,U	B,P,C	No	Python	GitHub	Funke et al [16]
sbm	Freq	D,U	B,P,C	Yes	R	CRAN	NA
sbm_canonical_mcmc	Bayes	U	B	No	C++	GitHub	Young et al [17]
sbm-cov	Freq	U	B	Yes	R	GitHub	Mu et al [18]
sbmr	Bayes	U	B,P	No	R	GitHub	NA
sbmSDP	Freq	U	B	No	R	CRAN	NA
SBMSplitMerge	Bayes	D,U	B,P,C	No	R	CRAN	Ludkin et al [19]
SparseBM	Freq	D,U	B	No	Python	GitLab	NA

TABLE S2 Online documentation for implementing available stochastic block model software packages. The year of the most recent update is provided in the last column.

Package	URL	Updated
anocva	https://cran.r-project.org/web/packages/anocva/index.html	2023
BipartiteSBM	https://github.com/junipertcy	2020
blockmodeling	https://cran.r-project.org/web/packages/blockmodeling/index.html	2023
blockmodels	https://cran.r-project.org/web/packages/blockmodels/index.html	2022
CommunityDetection	https://github.com/Jonas1312/community-detection-in-graphs	2017
dBlockmodeling	https://cran.r-project.org/web/packages/dBlockmodeling/index.html	2023
ESBM	https://github.com/danieledurante/ESBM/tree/master	2022
expSBM	https://cran.r-project.org/web/packages/expSBM/index.html	2022
graphon	https://cran.r-project.org/web/packages/graphon/index.html	2022
graph-tool	https://graph-tool.skewed.de	2024
greed	https://cran.r-project.org/web/packages/greed/index.html	2022
GREMLIN	https://cran.r-project.org/web/packages/gremlin/index.html	2022
igraph	https://cran.r-project.org/web/packages/igraph/index.html	2024
I-Louvain	https://www.dropbox.com/sh/j4aqitujiaifgq4/AAAAHOL3uIPYNWKoLpcAhOTPa	2014
missSBM	https://cran.r-project.org/web/packages/missSBM/index.html	2023
MODE-NET	http://www.lps.ens.fr/~krzakala/MODE_NET/	2014
noisySBM	https://cran.r-project.org/web/packages/noisySBM/index.html	2022
pysbm	https://github.com/funket/pysbm/tree/master/pysbm	2019
sbm	https://cran.r-project.org/web/packages/sbm/index.html	2024
sbm_canonical_mcmc	https://github.com/jg-you/sbm_canonical_mcmc	2019
sbm-cov	https://github.com/CongM/sbm-cov	2021
sbmr	https://github.com/tbilab/sbmr	2020
sbmSDP	https://cran.r-project.org/web/packages/sbmSDP/	2022
SBMSplitMerge	https://cran.r-project.org/web/packages/SBMSplitMerge/index.html	2022
SparseBM	https://gitlab.com/jbleger/sparsebm	2023

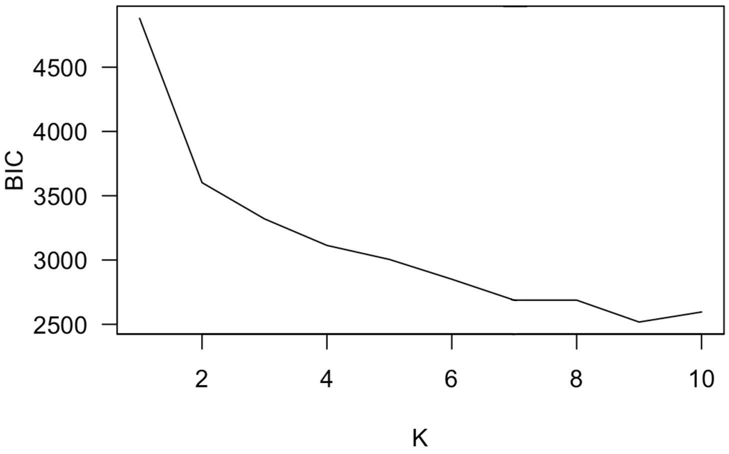

FIGURE S1 Scree plot of BIC for the rUTI dataset using Bayesian-SBM-MRF when $d = 1$.

TABLE S3 A comparison of the number of communities that selected genera have been assigned to using Bayesian-SBM-MRF when $d = 0$ and $d = 1$.

	Genus					
**Setting**	*Peptoniphilus*	*Facklamia*	*Bifidobacterium*	*Staphylococcus*	*Corynebacterium*	*Anaerococcus*
$d = 0$	2	2	2	2	4	4
$d = 1$	1	1	1	1	2	3

S2 — URINARY MICROBIOME DIVERSITY ANALYSIS

Our model encourages taxa from the same genus to be clustered together when $d > 0$, which reduces community diversity with respect to genus compared to when no taxonomic tree information is leveraged ($d = 0$). In other words, we expect some species of the same genus to belong to fewer communities when $d > 0$. In this section, we continue the real urinary microbiome data analysis from Section 5.1 of the main manuscript with an exploratory analysis of diversity with respect to genus within each community for Bayesian-SBM-MRF when $d = 0$ and $d = 1$ (our recommended setting).

Figure S2 illustrates both intra-community and intra-network nodal degree with respect to genus within each community for Bayesian-SBM-MRF when $d = 0$ and $d = 1$. From the plots, it can be observed that there is some difference in diversity with respect to genus within several communities. For example, some intra-community hubs have greater nodal degree when $d = 1$ compared to $d = 0$ indicating an increase in membership to the same community of species from the same genus. While diversity has appeared to decrease, most hubs in the plots where $d = 0$ appear to also be hubs in the plots where $d = 1$ (e.g., *Peptoniphilus*, *Corynebacterium*, *Prevotella*, and *Lactobacillus*). To quantify the diversity with respect to genus within each community, we calculated the Shannon index for both model settings. The Shannon index is the log transformation of the weighted geometric mean of proportion of species per genus in a

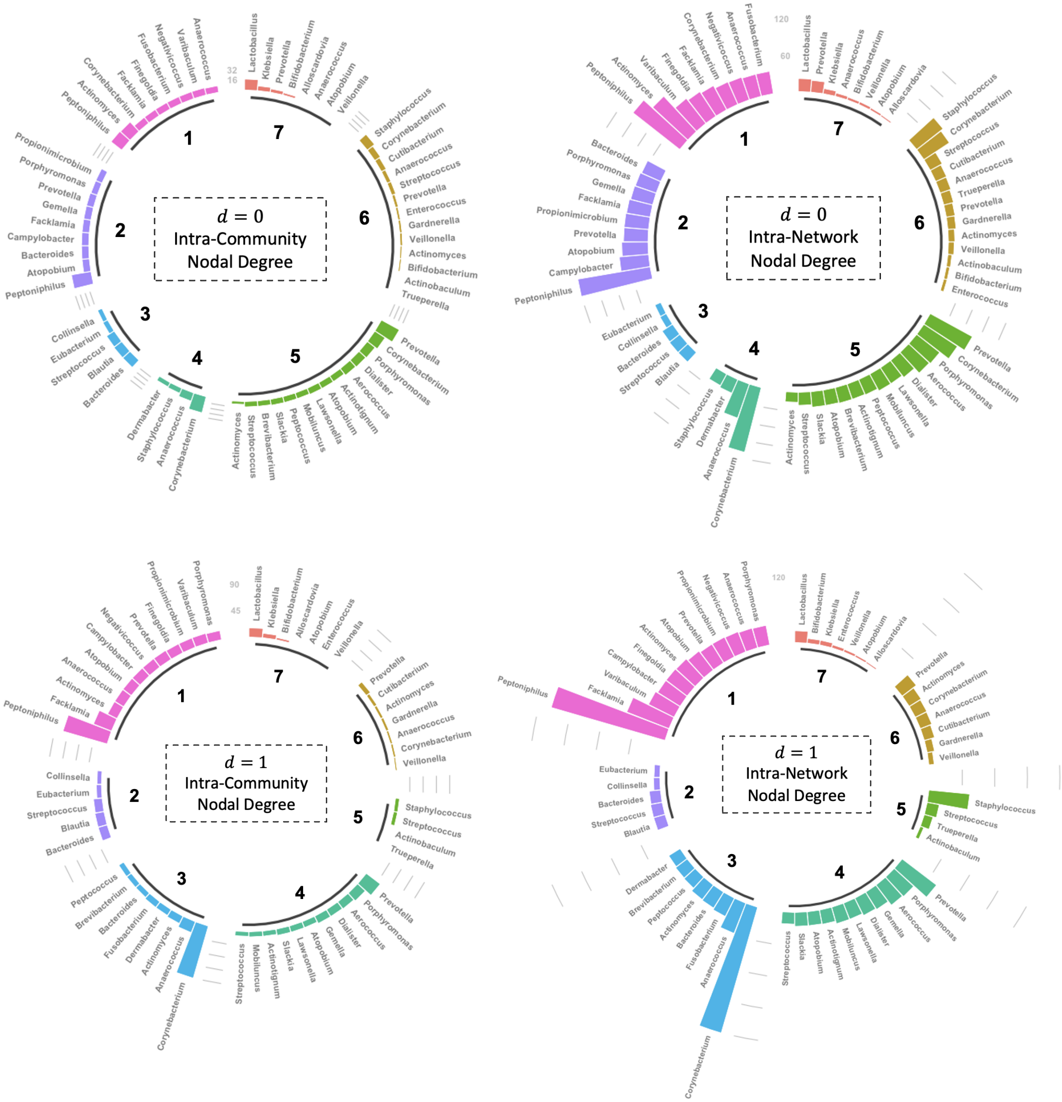

FIGURE S2 Circular bar plots of the intra-community and intra-network nodal degree with respect to genus within each community. The two figures at the top correspond to Bayesian-SBM-MRF when $d = 0$, which incorporates no taxonomic tree information; the two figures at the bottom correspond to Bayesian-SBM-MRF when $d = 1$, which incorporates taxonomic tree information.

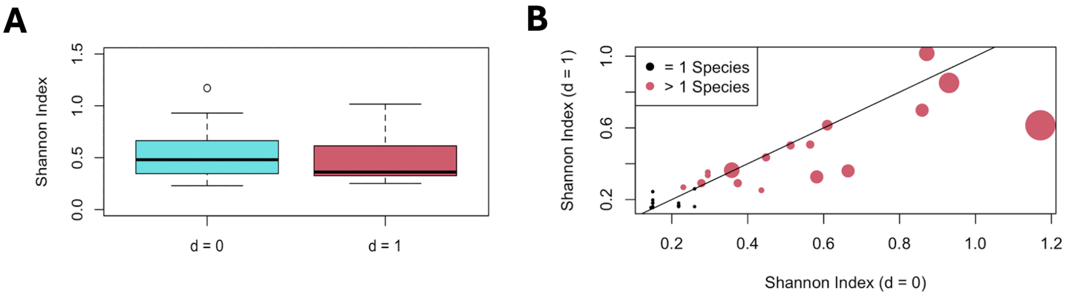

FIGURE S3 Plots of the Shannon entropies of the 41 genera from the rUTI data for Bayesian-SBM-MRF model settings $d = 0$ and $d = 1$. **A**: Boxplots of the Shannon entropies; **B**: Scatter plot of the Shannon entropies with the 45° line; each coordinate pair (x, y) gives the entropies of genus r for model settings $d = 0$ and $d = 1$, respectively; the size of points varies with respect to the number of species per genus.

given community.[20] In general, the Shannon index for genus r is denoted as H_r and calculated as

$$H_r = -\sum_{k=1}^{K} h_{kr} \log\left(h_{kr}\right)$$

where h_{kr} is the proportion of species belonging to the rth genus for $r = 1, \ldots, R$ in community k for $k = 1, \ldots, K$. In general, R Shannon entropies are calculated (one for each genus). For the real data, we calculated $R = 41$ Shannon entropies since there were 41 genera. Figure S3 compares the 41 Shannon entropies of the two model settings when $d = 0$ and $d = 1$. From Figure S3, we can observe that entropy decreases when switching from $d = 0$ to $d = 1$. First, from Figure S3A, the median Shannon entropy is lower for $d = 1$. Further, the maximum entropy when $d = 0$ is an outlier unlike the maximum entropy when $d = 1$. Second, in Figure S3B, each x and y correspond to the Shannon entropies of genus r when $d = 0$ and $d = 1$, respectively. The points tend to fall more below the 45° line than above it indicating lower diversity with respect to genus within each community when $d = 1$ compared to $d = 0$.

references

1. Vidal MC, Sato JR, Balardin JB, Takahashi DY, Fujita A. ANOCVA in R: a software to compare clusters between groups and its application to the study of autism spectrum disorder. Frontiers in Neuroscience 2017;11:16.

2. Žiberna A. Generalized blockmodeling of valued networks. Social Networks 2007;29(1):105–126.

3. Leger JB. Blockmodels: A R-package for estimating in Latent Block Model and Stochastic Block Model, with various probability functions, with or without covariates. arXiv preprint arXiv:160207587 2016;.

4. Brusco M, Doreian P, Steinley D. Deterministic blockmodelling of signed and two-mode networks: A tutorial with software and psychological examples. British Journal of Mathematical and Statistical Psychology 2021;74(1):34–63.

5. Legramanti S, Rigon T, Durante D, Dunson DB. Extended stochastic block models with application to criminal networks. The Annals of Applied Statistics 2022;16(4):2369.

6. Rastelli R, Fop M. A dynamic stochastic blockmodel for interaction lengths. arXiv preprint arXiv:190109828 2019;.

7. Orbanz P, Roy DM. Bayesian models of graphs, arrays and other exchangeable random structures. IEEE transactions on pattern analysis and machine intelligence 2014;37(2):437–461.

8. Peixoto TP. Bayesian stochastic blockmodeling. Advances in network clustering and blockmodeling 2019;p. 289–332.

9. Côme E, Jouvin N. greed: An R Package for Model-Based Clustering by Greedy Maximization of the Integrated Classification Likelihood. arXiv preprint arXiv:220414063 2022;.

10. Bar-Hen A, Barbillon P, Donnet S. Block models for generalized multipartite networks: Applications in ecology and ethnobiology. Statistical Modelling 2020;p. 1471082X20963254.

11. Schweinberger M, Luna P. HERGM: Hierarchical exponential-family random graph models. Journal of Statistical Software 2018;85:1–39.

12. Csardi G, Nepusz T, et al. The igraph software package for complex network research. InterJournal, Complex Systems 2006;1695(5):1–9.

13. Combe D, Largeron C, Géry M, Egyed-Zsigmond E. I-louvain: An attributed graph clustering method. In: Advances in Intelligent Data Analysis XIV: 14th International Symposium, IDA 2015, Saint Etienne. France, October 22-24, 2015. Proceedings 14 Springer; 2015. p. 181–192.

14. Barbillon P, Chiquet J, Tabouy T. missSBM: An r package for handling missing values in the stochastic block model. arXiv preprint arXiv:190612201 2019;.

15. Rebafka T, Roquain E, Villers F. Graph inference with clustering and false discovery rate control. arXiv preprint arXiv:190710176 2019;.

16. Funke T, Becker T. Stochastic block models: A comparison of variants and inference methods. PloS one 2019;14(4):e0215296.

17. Young JG, Desrosiers P, Hébert-Dufresne L, Laurence E, Dubé LJ. Finite-size analysis of the detectability limit of the stochastic block model. Physical Review E 2017;95(6):062304.

18. Mu C, Mele A, Hao L, Cape J, Athreya A, Priebe CE. On spectral algorithms for community detection in stochastic blockmodel graphs with vertex covariates. IEEE Transactions on Network Science and Engineering 2022;9(5):3373–3384.

19. Ludkin M. Inference for a generalised stochastic block model with unknown number of blocks and non-conjugate edge models. Computational Statistics & Data Analysis 2020;152:107051.

20. Tucker CM, Cadotte MW, Carvalho SB, Davies TJ, Ferrier S, Fritz SA, et al. A guide to phylogenetic metrics for conservation, community ecology and macroecology. Biological Reviews 2017;92(2):698–715.

21. Kass RE, Raftery AE. Bayes factors. Journal of the american statistical association 1995;90(430):773–795.
